# Association of piperacillin and vancomycin exposure on acute kidney injury during combination therapy

**DOI:** 10.1093/jacamr/dlad157

**Published:** 2024-01-22

**Authors:** Veena Venugopalan, Nicole Maranchick, Devorah Hanai, Yaima Jimenez Hernandez, Yuliya Joseph, Amanda Gore, Kathryn Desear, Charles Peloquin, Michael Neely, Timothy Felton, Bethany Shoulders, Mohammad Alshaer

**Affiliations:** Department of Pharmacy, UF Health Shands Hospital, Gainesville, FL, USA; Department of Pharmacy and Translational Research, University of Florida College of Pharmacy, Gainesville, FL, USA; Department of Pharmacy and Translational Research, University of Florida College of Pharmacy, Gainesville, FL, USA; Department of Pharmacy and Translational Research, University of Florida College of Pharmacy, Gainesville, FL, USA; Department of Pharmacy and Translational Research, University of Florida College of Pharmacy, Gainesville, FL, USA; Department of Pharmacy and Translational Research, University of Florida College of Pharmacy, Gainesville, FL, USA; Department of Pharmacy and Translational Research, University of Florida College of Pharmacy, Gainesville, FL, USA; Department of Pharmacy, UF Health Shands Hospital, Gainesville, FL, USA; Department of Pharmacy and Translational Research, University of Florida College of Pharmacy, Gainesville, FL, USA; Children's Hospital Los Angeles, Keck School of Medicine, University of Southern California, Los Angeles, CA, USA; Division of Immunology, Immunity to Infection and Respiratory Medicine, The University of Manchester, Manchester, UK; Department of Pharmacy, UF Health Shands Hospital, Gainesville, FL, USA; Department of Pharmacy and Translational Research, University of Florida College of Pharmacy, Gainesville, FL, USA; Department of Pharmacy and Translational Research, University of Florida College of Pharmacy, Gainesville, FL, USA

## Abstract

**Objectives:**

Acute kidney injury (AKI) is a well-documented adverse effect observed with piperacillin/tazobactam in combination with vancomycin. The pharmacokinetics of these antibiotics when given in combination have not been previously evaluated. The purpose of this study was to compare the exposure of vancomycin + piperacillin/tazobactam in patients with and without AKI.

**Methods:**

Ninety adult patients, who received at least 72 h of vancomycin + piperacillin/tazobactam combination therapy and had available serum concentrations of vancomycin and piperacillin were included in the study. Nephrotoxicity was defined as a 1.5-fold increase in serum creatinine within 7 days from baseline. Median daily AUCs were calculated in those with nephrotoxicity (vancomycin + piperacillin/tazobactam ‘N’) versus those without nephrotoxicity (vancomycin + piperacillin/tazobactam ‘WN’) during the first 7 days of combination therapy.

**Results:**

The overall incidence of AKI in those receiving vancomycin + piperacillin/tazobactam was 20% (18/90). The median daily vancomycin AUCs did not differ between the vancomycin + piperacillin/tazobactam ‘WN’ and vancomycin + piperacillin/tazobactam ‘N’ groups. Although not statistically significant, the median daily vancomycin AUCs in the vancomycin + piperacillin/tazobactam ‘N’ group were numerically greater on Day 5 and trended downwards thereafter. For the piperacillin group, the median daily AUCs did not vary between groups, except on Day 7 where the vancomycin + piperacillin/tazobactam ‘WN’ group had statistically greater median piperacillin AUC than the vancomycin + piperacillin/tazobactam ‘N’ group (*P* = 0.046).

**Conclusions:**

Utilizing serum creatinine-defined AKI, our study did not find any significant differences in vancomycin and piperacillin/tazobactam exposure between the groups with and without nephrotoxicity. These data indicate that vancomycin + piperacillin/tazobactam should not be avoided due to the risk of overexposure; instead, clinicians should continue to use these therapies cautiously.

## Background

Antibiotic-related renal toxicity accounts for one in four adverse events in hospitalized patients.^1^ Acute kidney injury (AKI) is associated with increased mortality, increased length of stay and excess hospital costs, and occurs more commonly in septic patients admitted to the ICU.^2,3^

Piperacillin/tazobactam in combination with vancomycin is a frequently used empirical antibiotic regimen in hospitalized patients due to the broad spectrum of activity. The components of the combination were ranked first and second among antibiotics administered to critical care patients in a point prevalence survey at 183 US hospitals.^4^ Nephrotoxicity is a well-documented adverse effect associated with vancomycin, particularly with earlier formulations due to the presence of impurities.^5^ Some risk factors for vancomycin-associated AKI are higher total daily doses, higher troughs, elevated 24 h AUC, prolonged treatment and concomitant use of nephrotoxins.^5,6^ Piperacillin/tazobactam has not been shown to increase the incidence of nephrotoxicity, although one study in critically ill patients showed delayed improvement in serum creatinine with piperacillin/tazobactam versus meropenem.^7,8^ The risk of AKI from the combination of vancomycin + piperacillin/tazobactam has been described in numerous observational studies and meta-analyses.^9–17^ Taken together, the data indicate a significant signal for nephrotoxicity with vancomycin + piperacillin/tazobactam, although the clinical and long-term impact of the combination are less well defined.^18^

In an era of growing antimicrobial resistance, utilizing first-line antibiotics such as vancomycin + piperacillin/tazobactam and preserving alternative antibiotics for more resistant, difficult-to-treat pathogens is imperative. Despite growing data describing the increased risk of AKI with vancomycin + piperacillin/tazobactam, the pharmacokinetics (PK) of these antibiotics when given in combination have not been studied. The purpose of this study is to compare the exposure of vancomycin + piperacillin/tazobactam in patients with and without nephrotoxicity.

## Methods

This was a single-centre, retrospective review between December 2017 and September 2022 at the University of Florida Health Shands Hospital, which is a 1162 bed tertiary academic medical centre in Gainesville, Florida. Patients were included in the study if they were ≥18 years old, received at least 72 h of vancomycin + piperacillin/tazobactam combination therapy and had available serum concentrations of vancomycin and piperacillin. Tazobactam concentration assays were not available. Patients were excluded if receiving renal replacement therapy at the start of vancomycin + piperacillin/tazobactam.

β-Lactam therapeutic drug monitoring was introduced into clinical usage at our institution in 2016. Total vancomycin and piperacillin concentrations were obtained as part of routine clinical care. Trough-based monitoring of vancomycin is the standard practice at this institution. Vancomycin dosing was performed based on the institutional policy. For piperacillin, steady-state random concentrations were obtained for continuous infusions; peak and trough concentrations were obtained for extended and intermittent infusions. Continuous infusions were administered over 24 h infusions, extended infusions over 3–4 h, and intermittent infusions over 30 min. Total plasma concentrations of piperacillin were measured in the Infectious Diseases Pharmacokinetics Lab (IDPL) at the University of Florida, using a validated LC with tandem MS assay. Vancomycin and piperacillin posterior predictions were generated using Pmetrics 1.9.7, and Phoenix WinNonlin (v8.3) was used to calculate AUCs. CL_CR_ was estimated using the Cockcroft–Gault formula.^19^

Patient demographics, comorbid conditions, laboratory values, drug dosing, drug concentrations and clinical course were extracted from the electronic medical records (Epic^®^ 2015 software; Verona, WI, USA). The study was approved by the Institutional Review Board (IRB) at the University of Florida.

### Outcomes and definitions

The definition of AKI was adapted from the Kidney Disease Improving Global Outcomes (KDIGO) clinical practice guidelines. Nephrotoxicity was defined as a 1.5-fold increase in serum creatinine within 7 days from baseline. AKI severity was stratified into three categories: KDIGO stage 1 (serum creatinine 1.5–1.9 times increased from baseline), KDIGO stage 2 (serum creatinine ≥2 times increased from baseline), and KDIGO stage 3 (serum creatinine 3 times increased from baseline or initiation of renal replacement therapy). Baseline was defined as the first day of concurrent vancomycin and piperacillin/tazobactam therapy. The primary outcome was to compare median daily AUCs in those with nephrotoxicity (vancomycin + piperacillin/tazobactam ‘N’) versus those without nephrotoxicity (vancomycin + piperacillin/tazobactam ‘WN’) during the first 7 days of combination therapy.

## Statistical analysis

Comparisons between the vancomycin + piperacillin/tazobactam ‘N’ and vancomycin + piperacillin/tazobactam ‘WN’ groups were performed using independent Student’s *t*-test for continuous data with normal distribution and chi-squared test or Fisher’s exact test for categorical data, as appropriate. The Mann–Whitney *U*-test was used to compare medians for continuous variables not normally distributed. Statistical analysis was performed on JMP Pro v16.0 (SAS Institute, Cary, NC, USA).

## Results

Ninety patients were included in the analysis. The incidence of AKI with vancomycin + piperacillin/tazobactam, based on the study definition, was 20% (18/90). Baseline patient demographics did not vary between vancomycin + piperacillin/tazobactam ‘N’ and vancomycin + piperacillin/tazobactam ‘WN’ groups (Table 1). Those with vancomycin + piperacillin/tazobactam ‘N’ had lower median serum creatinine on Day 1 of combination therapy versus vancomycin + piperacillin/tazobactam ‘WN’ (0.48 versus 0.79, *P* = 0.0017). The maximum median serum creatinine within 7 days of starting combination was greater in the vancomycin + piperacillin/tazobactam ‘N’ group compared with those in the vancomycin + piperacillin/tazobactam ‘WN’ group; however, this difference was not statistically significant (1.22 versus 0.92, *P* = 0.21). The median time to highest serum creatinine from starting combination therapy was 6.5 days (IQR 5–7) with vancomycin + piperacillin/tazobactam ‘N’ versus 3 days (IQR 1–3) with vancomycin + piperacillin/tazobactam ‘WN’ (*P* = 0.0003). Of the 18 patients who met the AKI definition, 12 (67%) were classified as KDIGO stage 1, 5 (33%) as KDIGO stage 2, and 1 (6%) as KDIGO stage 3. The patient with KDIGO stage 3 AKI was started on continuous veno-venous haemofiltration 5 days after starting vancomycin + piperacillin/tazobactam. The median duration of vancomycin or piperacillin/tazobactam therapy did not vary between groups. Additionally, the median duration of vancomycin + piperacillin/tazobactam combined therapy did not differ between those who did and did not develop AKI. Median daily piperacillin and vancomycin doses did not vary; however, more patients in the vancomycin + piperacillin/tazobactam ‘N’ group did not receive loading doses of vancomycin (Table 2). Appropriately obtained piperacillin and vancomycin concentrations were obtained in 75 (12 vancomycin + piperacillin/tazobactam ‘N’, 63 vancomycin + piperacillin/tazobactam ‘WN’) and 89 (18 vancomycin + piperacillin/tazobactam ‘N’, 71 vancomycin + piperacillin/tazobactam ‘WN’) patients, respectively. In comparison with the median vancomycin trough in the vancomycin + piperacillin/tazobactam ‘N’ group (9.3 mg/L, IQR 7.8–10.1), that in the vancomycin + piperacillin/tazobactam ‘WN’ group was statistically greater (12 mg/L, IQR 9.3−16.8) (*P* = 0.024). There were no differences noted in piperacillin trough concentrations between groups. In the vancomycin + piperacillin/tazobactam ‘N’ group, piperacillin/tazobactam dose adjustments were made in 72% (*n* = 13/17) of the cases following β-lactam concentrations. In 10 cases, a change was made from intermittent to continuous infusions of piperacillin/tazobactam. Two patients had their total daily dose decreased, and in one additional case, the total daily dose was increased. Dose adjustments for vancomycin were made in all but two patients in the vancomycin + piperacillin/tazobactam ‘N’ group. In 10 of these cases, the vancomycin dose and/or dosing frequency was increased.

**Table 1.** Patient demographics

Characteristic	All patients *n* = 90	VPT-N *n* = 18	VPT-WN *n* = 72	*P*
Age (years), median (IQR)	41.5 (29–58)	37 (24.8–57)	45 (29–62)	0.28
Male sex, *n* (%)	52 (58)	12 (67)	40 (56)	0.39
BMI (kg/m^2^), median (IQR)	24.6 (21.6–28.2)	23.6 (19.4–26)	25.2 (21.6–29.7)	0.11
Race, *n* (%)				
Black	11 (12)	4 (22)	7 (10)	0.15
White	71 (79)	12 (67)	59 (82)	0.11
Latino	2 (2)	0	2 (3)	0.47
Other	6 (7)	2 (11)	4 (6)	0.55
Admitted from, *n* (%)				
Home	52 (58)	7 (39)	45 (63)	0.14
Nursing home	4 (4)	0	4 (6)	0.31
Hospital-to-hospital transfer	34 (38)	11 (61)	23 (32)	0.15
Charlson comorbidity index, median (IQR)	2 (0–4)	1 (0–2)	2 (0–4)	0.12
ICU during combination, *n* (%)	37 (41)	9 (50)	28 (39)	0.39
Mechanical ventilation, *n* (%)	21 (23)	5 (28)	16 (22)	0.62
Nephrotoxins, *n* (%)				
Vasopressors	23 (26)	6 (33)	17 (24)	0.38
Aminoglycosides	5 (6)	1 (6)	4 (6)	1.00
Angiotensin-converting enzyme inhibitor (ACEI)	5 (6)	1 (6)	4 (6)	1.00
Angiotensin II receptor blocker	1(1)	0	1 (1)	1.00
Diuretics	28 (31)	3 (17)	25 (35)	0.17
IV contrast	34 (38)	5 (28)	29 (40)	0.42
In-hospitality mortality, *n* (%)	7 (8)	2 (11)	5 (7)	0.55
Hospital length of stay (days), median (IQR)	14 (10–26.5)	21.5 (11.8–33.5)	14 (9–25)	0.14
Serum creatinine on the first day of combination therapy (mg/dL), median (IQR)	0.75 (0.57–1.02)	0.48 (0.39–0.79)	0.79 (0.62–1.08)	**0**.**0017**
Highest serum creatinine within 7 days of initiating combination therapy (mg/dL), median (IQR)	0.93 (0.71–1.25)	1.22 (0.70–1.62)	0.92 (0.71–1.16)	0.21
Creatinine clearance on the first day of combination therapy (mL/min), median (IQR)	96.6 (66.5–128.9)	135.6 (96.9–214.4)	89.9 (63.8–120.1)	**0**.**0016**
Highest creatinine clearance within 7 days of initiating combination therapy (mL/min), median (IQR)	76.7 (54.7–97.6)	65.8 (50.8–109)	80.3 (54.9–96.9)	0.44
Time to highest serum creatinine from starting combination therapy (days), median (IQR)	3 (1–6)	6.5 (5–7)	3 (1–6)	**0**.**0003**
Duration of piperacillin/tazobactam (days), median (IQR)	9 (6.8–13)	11 (8.5–15)	9 (5.3–12)	0.025
Duration of vancomycin (days), median (IQR)	8 (5–13)	8.5 (4.8–14)	8 (5–11)	0.59
Duration of overlap (days), median (IQR)	7 (5–11)	8 (6–13)	6 (5–10)	0.085

VPT-N, vancomycin + piperacillin/tazobactam with nephrotoxicity; VPT-WN, vancomycin + piperacillin/tazobactam with nephrotoxicity. Bold type indicates statistical significance.

**Table 2.** Dosing and concentrations

Dosing	All patients *n* = 90	VPT-N *n* = 18	VPT-WN *n* = 72	*P*
Piperacillin daily dose (g), median (IQR)	16 (12–16)	16 (12–16)	16 (12–16)	0.11
Vancomycin loading dose, *n* (%)	58 (64)	9 (50)	49 (68)	0.15
Vancomycin daily dose (g), median (IQR)	2.3 (1.5–3)	2.5 (1.5–3.1)	2 (1.5–3)	0.23
Piperacillin/tazobactam infusion				0.91
Intermittent	49 (54)	9 (50)	40 (56)	
Extended	27 (30)	6 (33)	21 (29)	
Continuous	14 (16)	3 (17)	11 (15)	
Vancomycin trough (μg/mL), median (IQR)^a^	11.1 (8.7–15.4)	9.3 (7.8–10.1)	12 (9.3–16.8)	**0.024**
Piperacillin trough (μg/mL), median (IQR)^b^	24.3 (12.7–41.3)	19.3 (10–33.4)	24.4 (13.3–46.1)	0.25

VPT-N, vancomycin + piperacillin/tazobactam with nephrotoxicity; VPT-WN, vancomycin + piperacillin/tazobactam without nephrotoxicity. Bold type indicates statistical significance.

^a^ *n* = 89 (18 VPT-N, 71 VPT-WN).

^b^ *n* = 75 (12 VPT-N, 63 VPT-WN).

**Figure 1.**
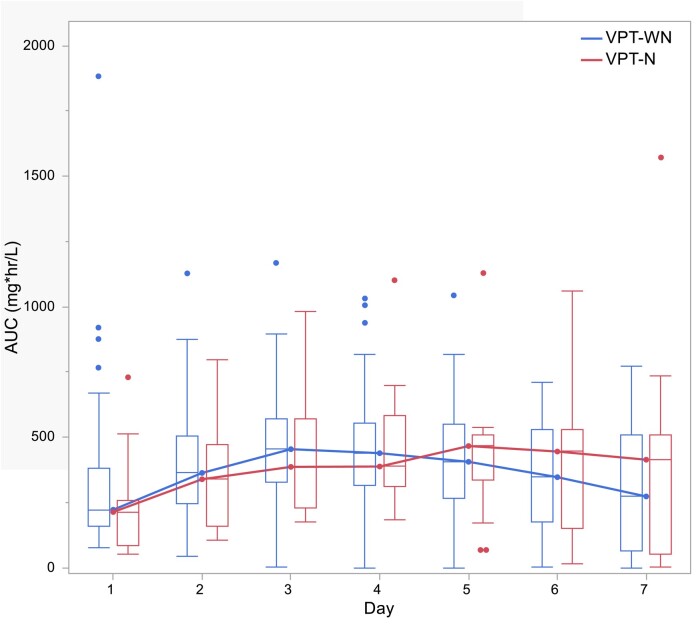
Daily median vancomycin AUCs. Figure was generated using statistical software. VPT-N, vancomycin + piperacillin/tazobactam with nephrotoxicity; VPT-WN, vancomycin + piperacillin/tazobactam with nephrotoxicity.

**Figure 2.**
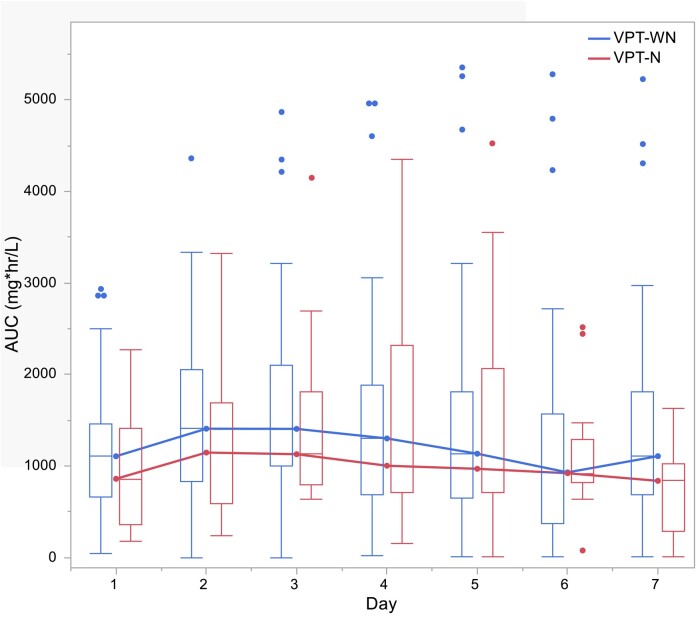
Daily median piperacillin AUCs. Figure was generated using statistical software. VPT-N, vancomycin + piperacillin/tazobactam with nephrotoxicity; VPT-WN, vancomycin + piperacillin/tazobactam with nephrotoxicity.

In terms of the primary outcome, the median daily vancomycin AUCs did not differ between the vancomycin + piperacillin/tazobactam ‘WN’ and vancomycin + piperacillin/tazobactam ‘N’ groups (Table 3). The median daily AUCs in the vancomycin + piperacillin/tazobactam ‘N’ group were numerically greater on Day 5 and trended downwards thereafter (Figure 1); however, this difference was not statistically significant. For the piperacillin group, the median daily AUCs did not vary between groups. In fact, piperacillin AUCs tracked closely between groups (Figure 2) except on Day 7 where the vancomycin + piperacillin/tazobactam ‘WN’ group had statistically greater median AUC compared with the vancomycin + piperacillin/tazobactam ‘N’ group (*P* = 0.046). Total vancomycin and piperacillin AUCs over the entire combination period did not vary between groups.

**Table 3.** Daily vancomycin and piperacillin AUCs

AUC (mg·h/L), median (IQR)	VPT-N *n* = 18	VPT-WN *n* = 72	*P*
Piperacillin AUC Day 1	859.2 (358.8–1407.3)	1105.8 (664.4–1463.9)	0.29
Piperacillin AUC Day 2	1146 (591.1–1685.8)	1406.7 (827.4–2054.7)	0.27
Piperacillin AUC Day 3	1128 (789–1815)	1404.9 (1004.1–2103.6)	0.31
Piperacillin AUC Day 4	1002.1 (705.3–2314.6)	1300.4 (690.2–1881.2)	0.79
Piperacillin AUC Day 5	968.4 (707.9–2066.5)	1133.5 (643.2–1809.8)	1.00
Piperacillin AUC Day 6	920.2 (812–1291.5)	929.5 (373.3–1562.6)	0.50
Piperacillin AUC Day 7	836.8 (286.1–1026.6)	1106.5 (690–1804.4)	**0.046**
Total piperacillin AUC for the combination period	8585.2 (4915.6–1218.1)	8375.9 (5343.1–13 659.4)	0.73
Vancomycin AUC Day 1	213.3 (82.8–256.4)	221.9 (159.6–380.7)	0.13
Vancomycin AUC Day 2	338.8 (158.1–471.3)	363 (245.8–505.7)	0.33
Vancomycin AUC Day 3	386.1 (228.1–568.6)	454 (327–570.6)	0.48
Vancomycin AUC Day 4	387.5 (311.7–580.8)	438.7 (316.7–555.2)	0.79
Vancomycin AUC Day 5	466 (336.8–510.3)	405.6 (265.2–549.5)	0.80
Vancomycin AUC Day 6	445 (151.5–530.7)	346.8 (173.6–527.2)	0.72
Vancomycin AUC Day 7	413.7 (51.8–509.7)	272.9 (65.8–510)	0.85
Total vancomycin AUC for the combination period	3442.887 (1951.7–4973.1)	2856.1 (1870.4–4688.4)	0.55

VPT-N, vancomycin + piperacillin/tazobactam with nephrotoxicity; VPT-WN, vancomycin + piperacillin/tazobactam without nephrotoxicity. Bold type indicate statistical significance.

## Discussion

The incidence of AKI with vancomycin + piperacillin/tazobactam is highly variable, with rates reported between 7.4% and 52.7% depending on the population studied.^20^ The variability in AKI rates may be attributed to the discrepancy in definitions for AKI [Acute Kidney Injury Network (AKIN), KDIGO, or risk, injury, failure, loss, end-stage kidney disease (RIFLE)] as well as the heterogeneity of the populations studied. Lack of a standardized definition for drug-induced kidney injury (DIKD) has prompted researchers to develop a classification system based on novel biomarkers (both functional and damage related) and common mechanisms of nephrotoxicity.^21^ Until novel biomarkers of kidney function are widely used in the clinical setting, these definitions may not be fully implemented. Published studies indicate lower rates of AKI with vancomycin + piperacillin/tazobactam if critically and non-critically ill patients are combined, and higher rates when critically ill patients are evaluated alone.^19^ In our study, which was comprised of a mixed population of critically and non-critically ill patients, the incidence of AKI was 20%. This is within the range reported elsewhere. Several studies have compared the incidence of AKI with vancomycin + piperacillin/tazobactam to that of vancomycin in combination with other β-lactams. Buckley and colleagues^22^ performed a multicentre, retrospective, propensity score-matched cohort study to compare the incidence of AKI of vancomycin in combination with piperacillin/tazobactam, cefepime or meropenem. The overall incidence of AKI in the vancomycin + piperacillin/tazobactam group was 21.9% versus 16.8% in the vancomycin + cefepime/meropenem groups combined (*P* = 0.068). Similar to our study, where 67% of patients (*n* = 12/18) were determined to be KDIGO stage 1, in the aforementioned study, the majority of patients developed mild AKI (KDIGO stage I) with significantly higher rates observed with vancomycin + piperacillin/tazobactam than with vancomycin + cefepime/meropenem. The onset of AKI in a matched cohort study was 5 days in the vancomycin + piperacillin/tazobactam group compared with 8 days in the vancomycin + cefepime group (*P* = 0.0001).^22^ This finding was consistent with our results where the median time to onset of AKI was 6.5 days. Shorter onset to AKI as early as 3 days with vancomycin + piperacillin/tazobactam when compared with vancomycin + cefepime/meropenem have been reported.^23–25^

The mechanism of nephrotoxicity with vancomycin + piperacillin/tazobactam has not been well defined. Vancomycin and piperacillin can independently cause nephrotoxicity. The primary mechanism of vancomycin-induced AKI is oxidative stress, which leads to mitochondrial dysfunction and cellular apoptosis.^26^ There is also an allergic component to the pathomechanism associated with vancomycin-associated AKI. Vancomycin-induced nephrotoxicity can be caused by either acute tubular necrosis (ATN) or acute interstitial nephritis (AIN). AIN is also a well-known adverse effect of β-lactams. Kidney biopsies have confirmed AIN with piperacillin monotherapy. The lower incidence of AKI with vancomycin+cefepime compared to vancomycin + piperacillin/tazobactam is purported to be due to the decreased risk of inducing AIN with fourth-generation cephalosporins compared with piperacillin. Another mechanism of nephrotoxicity is via the organic anion transporters (OATs) located on the proximal tubular basolateral membranes. Piperacillin, tazobactam and vancomycin are excreted in the urine via OAT1 and OAT3.^27^ Since these transporters are also shown to mediate the transit of creatinine from peritubular circulation to tubular cells, the concomitant use of vancomycin + piperacillin/tazobactam results in the accumulation of serum creatinine. In addition to this, vancomycin inhibits mRNA expression of OAT1 and OAT3, which further contributes to the diminished excretion of creatinine.

Most studies evaluating AKI rely on serum creatinine as a surrogate for glomerular function, even though it is widely recognized as an unreliable indicator of renal injury. Glomerular filtration rates (GFRs) can drop as much as 50% before changes in creatinine are detected.^28^ Due to the limitations in the use of serum creatinine as an indicator of renal function, KIM-1, albumin, total protein, β2-microglobulin, cystatin C, clusterin and trefoil factor-3 are novel sensitive biomarkers capable of detecting kidney damage earlier than blood urea nitrogen (BUN) and creatinine levels.^29,30^ In an animal study evaluating the synergistic toxicity of vancomycin + piperacillin/tazobactam, urinary KIM-1 and clusterin increased with vancomycin monotherapy on Days 1, 2 and 3 compared with saline and only after 3 days of treatment with vancomycin + piperacillin/tazobactam compared with saline.^31^ Another translational animal model indicated a decline in GFR and an increase in KIM-1 in those rats that received vancomycin alone compared with those that received piperacillin/tazobactam alone or vancomycin + piperacillin/tazobactam.^32^ These results indicate that the AKI from vancomycin + piperacillin/tazobactam was not worse than that with vancomycin monotherapy. The kinetics of urinary biomarkers associated with AKI [tissue inhibitor of metalloproteinase-2 (TIMP-2) and insulin-like growth factor binding protein (IGFBP7)], were evaluated following renal insults such as surgery or drug exposure.^33^ This study concluded that exposure to multiple nephrotoxins during critical illness is common and cumulative renal insults increase the risk of AKI. In terms of biomarker kinetics, urinary [TIMP-2]·[IGFBP7] levels were elevated on the first day of vancomycin and/or piperacillin/tazobactam exposure, peaked the following day, and subsequently declined. Unfortunately, biomarker concentrations in those who received vancomycin + piperacillin/tazobactam combination versus monotherapy were not compared. Also, because the exact time of vancomycin and piperacillin/tazobactam administration was not reported, it was not possible to determine whether the increase in urinary biomarkers occurred before or after the time of exposure. Miano *et al.*^34^ conducted the first prospective clinical study to evaluate changes in creatinine and cystatin C concentrations after initiation of either vancomycin+piperacillin/tazobactam or vancomycin+cefepime. vancomycin+piperacillin/tazobactam was associated with a higher percentage increase in creatinine at Day 2 and higher incidence of creatinine-defined AKI. Despite the higher incidence of AKI, vancomycin + piperacillin/tazobactam was not associated with changes in cystatin C or BUN. These findings indicate that the increase in creatinine-defined AKI without changes in other urinary biomarkers likely represents pseudotoxicity. A similar pseudo-elevation of serum creatinine is observed with trimethoprim/sulfamethoxazole because it interferes with tubular excretion of creatinine without compromising the GFR.^35^

In our study, median daily AUCs did not vary for either vancomycin or piperacillin/tazobactam, except in the case of piperacillin/tazobactam where there was statistically greater median AUC in the vancomycin + piperacillin/tazobactam ‘WN’ group at Day 7. These findings differ from a recent investigation of critically ill paediatric patients, which showed that those with piperacillin-associated AKI had greater estimated AUC and the highest *C*_min_ in the first 24 h.^36^ Unfortunately, this study did not also evaluate concomitant vancomycin exposure in those patients with AKI, therefore the impact of combination therapy on AUC cannot be deduced. Kiley *et al.*^37^ investigated the incidence of AKI with vancomycin+piperacillin/tazobactam versus vancomycin+cefepime/meropenem. This study varied from those previously published due to the utilization of an AUC-based strategy to estimate vancomycin exposure between groups. Although the results showed a higher incidence of AKI with vancomycin+pipercillin/tazobactam versus vancomycin+cefepime/meropenem, vancomycin AUCs nevertheless did not vary between groups. It should be noted that in our study, the overall median vancomycin troughs and AUC were low. This observation could be attributed to lower median daily vancomycin doses in both groups as well as reduced use of loading doses. These findings warrant review of the institutional vancomycin dosing protocols.

This study is not without limitations. The retrospective study design and small sample size means that causality of AKI with vancomycin + piperacillin/tazobactam cannot be fully established. The study definition for AKI relied on serum creatinine, which as previously discussed is an unreliable measure of kidney function. Urine output or novel kidney function biomarkers, which are helpful in the early detection of AKI, were unavailable. Nephrotoxin exposure was evaluated as a binary variable. Therefore, the perceived nephrotoxic potential, number of doses and duration of nephrotoxin use was not evaluated. Use of a single trough for AUC estimation, particularly for vancomycin, might be suboptimal and may present the risk of model mis-specification. Lastly, we measured total piperacillin plasma concentrations. We acknowledge that antibiotic protein binding in critically ill patients can be highly variable due to factors such as hypoalbuminaemia, and renal and hepatic insufficiency.

### Conclusions

To our knowledge, this is the first study to evaluate vancomycin and piperacillin/tazobactam exposures in patients receiving combination therapy. With nephrotoxicity and serum creatinine-defined AKI according to accepted criteria (KDIGO), our study did not find any significant differences in vancomycin and piperacillin/tazobactam exposure between the group with and without nephrotoxicity. The lack of correlation between AKI and the AUCs of vancomycin and piperacillin, both renally excreted antibiotics, was either due to the mild AKI in our population (KDIGO Stages 1 and 2 only) or mechanisms of creatinine elevation unrelated to GFR, such as tubular transporter inhibition. Further studies that incorporate therapeutic drug monitoring or model-informed precision dosing with urinary biomarkers are needed to elucidate the clinical significance of the nephrotoxicity with this common antibiotic combination. Until then, this evidence suggests that clinicians may continue using vancomycin and piperacillin/tazobactam concomitantly when indicated but should do so while monitoring renal function and antibiotic concentrations.
